# 2-Amino-4,6-dimethyl­pyrimidine–benzoic acid (1/1)

**DOI:** 10.1107/S1600536809020649

**Published:** 2009-06-17

**Authors:** A-Lan Meng, Jun-E Huang, Bin Zheng, Zhen-Jiang Li

**Affiliations:** a Qingdao University of Science and Technology, Qingdao 266061, People’s Republic of China

## Abstract

The crystal of the title compound, C_6_H_9_N_3_·C_7_H_6_O_2_, contains tetra­meric hydrogen-bonded units comprising a central pair of 2-amino­pyrimidine mol­ecules linked across a centre of inversion by N—H⋯N hydrogen bonds and two pendant benzoic acid mol­ecules attached through N—H⋯O and O—H⋯N hydrogen bonds. These hydrogen-bonded units are arranged into layers in (002).

## Related literature

For the biological activity of pyrimidine and amino­pyrimidine derivatives, see: Hunt *et al.* (1980 ▶); Baker & Santi (1965 ▶). For related structures, see: Skovsgaard & Bond (2009 ▶); Fun *et al.* (2006 ▶); Wang *et al.* (2007 ▶); Schwalbe & Williams (1982 ▶); Hu *et al.* (2002 ▶); Chinnakali *et al.* (1999 ▶).
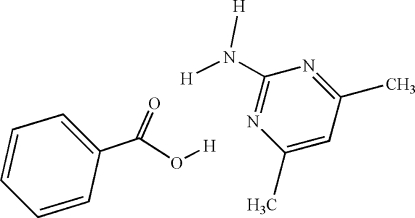


## Experimental

### 

#### Crystal data

C_6_H_9_N_3_·C_7_H_6_O_2_
*M* *_r_* = 245.28
Monoclinic, 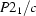

*a* = 6.7019 (9) Å
*b* = 7.6466 (10) Å
*c* = 25.285 (3) Å
β = 91.360 (2)°
*V* = 1295.4 (3) Å^3^
*Z* = 4
Mo *K*α radiation
μ = 0.09 mm^−1^
*T* = 295 K
0.18 × 0.15 × 0.10 mm

#### Data collection

Bruker SMART CCD diffractometer
Absorption correction: multi-scan (*SADABS*; Sheldrick, 1996 ▶) *T*_min_ = 0.985, *T*_max_ = 0.991
6594 measured reflections
2273 independent reflections
1228 reflections with *I* > 2σ(*I*)
*R*_int_ = 0.104

#### Refinement

*R*[*F* ^2^ > 2σ(*F* ^2^)] = 0.053
*wR*(*F* ^2^) = 0.137
*S* = 1.01
2273 reflections
167 parameters
H-atom parameters constrained
Δρ_max_ = 0.22 e Å^−3^
Δρ_min_ = −0.19 e Å^−3^

### 

Data collection: *SMART* (Bruker, 2001 ▶); cell refinement: *SAINT* (Bruker, 2001 ▶); data reduction: *SAINT*; program(s) used to solve structure: *SHELXS97* (Sheldrick, 2008 ▶); program(s) used to refine structure: *SHELXL97* (Sheldrick, 2008 ▶); molecular graphics: *SHELXTL* (Sheldrick, 2008 ▶); software used to prepare material for publication: *SHELXTL*.

## Supplementary Material

Crystal structure: contains datablocks global, I. DOI: 10.1107/S1600536809020649/bi2374sup1.cif

Structure factors: contains datablocks I. DOI: 10.1107/S1600536809020649/bi2374Isup2.hkl

Additional supplementary materials:  crystallographic information; 3D view; checkCIF report

## Figures and Tables

**Table 1:** Hydrogen-bond geometry (Å, °)

*D*—H⋯*A*	*D*—H	H⋯*A*	*D*⋯*A*	*D*—H⋯*A*
O1—H1⋯N1	0.82	1.82	2.606 (2)	160
N3—H3*A*⋯O2	0.86	2.16	3.003 (3)	168
N3—H3*B*⋯N2^i^	0.86	2.25	3.098 (3)	169

Symmetry code: (i) 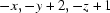.

**Fig. 1.**
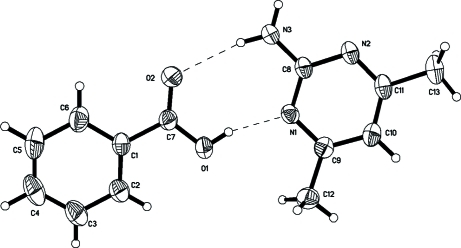
Molecular structure with displacement ellipsoids drawn at the 30% probability level for non-H atoms. Dashed lines denote hydrogen bonds.

**Fig. 2.**
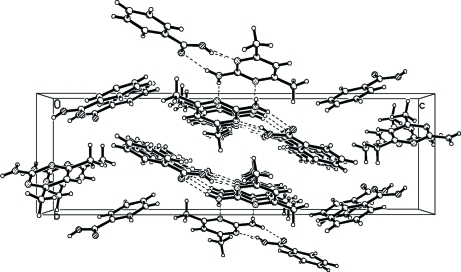
Packing diagram showing one layer of molecules connected by N—H···O and O—H···N hydrogen bonds (dashed lines).

## supplementary crystallographic information

### Comment

Pyrimidine and aminopyrimidine derivatives are biologically important compounds as they occur in nature as components of nucleic acids. Some aminopyrimidine derivatives are used as antifolate drugs (Hunt *et al.*, 1980; Baker & Santi, 1965). The crystal structures of aminopyrimidine derivatives (Schwalbe & Williams, 1982), aminopyrimidine carboxylates (Hu *et al.*, 2002) and co-crystal structures (Chinnakali *et al.*, 1999; Skovsgaard & Bond, 2009) have been reported.

The title compound (Fig. 1) was obtained as the product of an attempted synthesis of benzoic acid and 2-amino-4,6-dimethylpyrimidine in acetone. The bond lengths and angles in the pyrimidine ring and phenyl ring are generally normal (Fun *et al.*, 2006). The molecules associate through O—H···N, N—H···O and N—H···N hydrogen bonds into centrosymmetic tetrameric units. These units pack into stacked layers in the (002) planes (Fig. 2).

### Experimental

Single crystals of the title compound were obtained by reaction of benzoic acid (0.2 mmol) and 2-amino-4,6-dimethylpyrimidine (0.2 mmol) in refluxing acetone (50 ml). Single crystals suitable for X-ray analysis were obtained by recrystallization from ethanol solution at room temperature.

### Refinement

H atoms were fixed geometrically and allowed to ride on their attached atoms, with N—H = 0.86 Å, C—H = 0.93 or 0.96 Å, and with *U*_iso_(H) = 1.5 *U*_eq_(C) (for CH_3_) or 1.2 *U*_eq_(C) (for CH_2_, aromatic CH and NH_2_).

### Crystal data

**Table:**

C_6_H_9_N_3_·C_7_H_6_O_2_	*F*(000) = 520
*M**_r_* = 245.28	*D*_x_ = 1.258 Mg m^−^^3^
Monoclinic, *P*2_1_/*c*	Mo *K*α radiation, λ = 0.71073 Å
Hall symbol: -P 2ybc	Cell parameters from 167 reflections
*a* = 6.7019 (9) Å	θ = 1.6–25.0°
*b* = 7.6466 (10) Å	µ = 0.09 mm^−^^1^
*c* = 25.285 (3) Å	*T* = 295 K
β = 91.360 (2)°	Block, colourless
*V* = 1295.4 (3) Å^3^	0.18 × 0.15 × 0.10 mm
*Z* = 4	

### Data collection

**Table:**

Bruker SMART CCD diffractometer	2273 independent reflections
Radiation source: fine-focus sealed tube	1228 reflections with *I* > 2σ(*I*)
graphite	*R*_int_ = 0.104
φ and ω scans	θ_max_ = 25.0°, θ_min_ = 1.6°
Absorption correction: multi-scan (*SADABS*; Sheldrick, 1996)	*h* = −7→7
*T*_min_ = 0.985, *T*_max_ = 0.991	*k* = −9→9
6594 measured reflections	*l* = −22→30

### Refinement

**Table:**

Refinement on *F*^2^	Secondary atom site location: difference Fourier map
Least-squares matrix: full	Hydrogen site location: inferred from neighbouring sites
*R*[*F*^2^ > 2σ(*F*^2^)] = 0.053	H-atom parameters constrained
*wR*(*F*^2^) = 0.137	*w* = 1/[σ^2^(*F*_o_^2^) + (0.0352*P*)^2^ + 0.012*P*] where *P* = (*F*_o_^2^ + 2*F*_c_^2^)/3
*S* = 1.01	(Δ/σ)_max_ < 0.001
2273 reflections	Δρ_max_ = 0.22 e Å^−^^3^
167 parameters	Δρ_min_ = −0.19 e Å^−^^3^
0 restraints	Extinction correction: *SHELXL*, Fc^*^=kFc[1+0.001xFc^2^λ^3^/sin(2θ)]^-1/4^
Primary atom site location: structure-invariant direct methods	Extinction coefficient: 0.0077 (16)

### Special details

**Table:**

Geometry. All e.s.d.'s (except the e.s.d. in the dihedral angle between two l.s. planes) are estimated using the full covariance matrix. The cell e.s.d.'s are taken into account individually in the estimation of e.s.d.'s in distances, angles and torsion angles; correlations between e.s.d.'s in cell parameters are only used when they are defined by crystal symmetry. An approximate (isotropic) treatment of cell e.s.d.'s is used for estimating e.s.d.'s involving l.s. planes.
Refinement. Refinement of *F*^2^ against ALL reflections. The weighted *R*-factor *wR* and goodness of fit *S* are based on *F*^2^, conventional *R*-factors *R* are based on *F*, with *F* set to zero for negative *F*^2^. The threshold expression of *F*^2^ > σ(*F*^2^) is used only for calculating *R*-factors(gt) *etc*. and is not relevant to the choice of reflections for refinement. *R*-factors based on *F*^2^ are statistically about twice as large as those based on *F*, and *R*- factors based on ALL data will be even larger.

### Fractional atomic coordinates and isotropic or equivalent isotropic displacement parameters (Å^2^)

**Table:**

	*x*	*y*	*z*	*U*_iso_*/*U*_eq_	
O1	0.6583 (2)	0.6591 (2)	0.41923 (6)	0.0678 (5)	
H1	0.5870	0.7100	0.4402	0.102*	
O2	0.3940 (3)	0.6933 (3)	0.36625 (7)	0.0787 (6)	
N1	0.4633 (3)	0.7645 (2)	0.50129 (7)	0.0482 (5)	
N2	0.1976 (3)	0.9074 (2)	0.54612 (8)	0.0560 (6)	
N3	0.1836 (3)	0.8632 (2)	0.45633 (8)	0.0646 (6)	
H3A	0.2332	0.8250	0.4275	0.078*	
H3B	0.0689	0.9137	0.4557	0.078*	
C1	0.6891 (4)	0.5714 (3)	0.33125 (9)	0.0540 (6)	
C2	0.8886 (4)	0.5334 (3)	0.34037 (10)	0.0662 (7)	
H2	0.9484	0.5593	0.3730	0.079*	
C3	0.9993 (5)	0.4573 (4)	0.30126 (13)	0.0813 (9)	
H3	1.1337	0.4326	0.3075	0.098*	
C4	0.9124 (6)	0.4184 (4)	0.25352 (13)	0.0892 (10)	
H4	0.9866	0.3641	0.2276	0.107*	
C5	0.7167 (6)	0.4587 (4)	0.24346 (11)	0.0931 (10)	
H5	0.6586	0.4332	0.2106	0.112*	
C6	0.6032 (4)	0.5382 (4)	0.28248 (10)	0.0754 (8)	
H6	0.4708	0.5683	0.2754	0.090*	
C7	0.5664 (4)	0.6473 (3)	0.37388 (10)	0.0542 (7)	
C8	0.2837 (4)	0.8443 (3)	0.50166 (10)	0.0497 (6)	
C9	0.5618 (3)	0.7431 (3)	0.54739 (9)	0.0522 (6)	
C10	0.4805 (4)	0.8019 (3)	0.59401 (10)	0.0620 (7)	
H10	0.5481	0.7868	0.6262	0.074*	
C11	0.2973 (4)	0.8831 (3)	0.59146 (10)	0.0579 (7)	
C12	0.7598 (4)	0.6537 (3)	0.54573 (11)	0.0699 (8)	
H12A	0.7401	0.5309	0.5397	0.105*	
H12B	0.8303	0.6706	0.5788	0.105*	
H12C	0.8361	0.7022	0.5176	0.105*	
C13	0.1975 (4)	0.9484 (3)	0.64045 (10)	0.0793 (9)	
H13A	0.1886	1.0737	0.6393	0.119*	
H13B	0.2743	0.9136	0.6712	0.119*	
H13C	0.0658	0.8996	0.6421	0.119*	

### Atomic displacement parameters (Å^2^)

**Table:**

	*U*^11^	*U*^22^	*U*^33^	*U*^12^	*U*^13^	*U*^23^
O1	0.0593 (11)	0.0969 (15)	0.0473 (11)	0.0084 (10)	0.0053 (9)	−0.0141 (10)
O2	0.0568 (12)	0.1232 (16)	0.0564 (12)	0.0167 (11)	0.0055 (9)	−0.0012 (10)
N1	0.0425 (11)	0.0546 (12)	0.0480 (12)	−0.0031 (9)	0.0092 (9)	−0.0005 (9)
N2	0.0584 (13)	0.0594 (13)	0.0510 (13)	−0.0063 (10)	0.0179 (10)	−0.0042 (11)
N3	0.0552 (13)	0.0919 (16)	0.0472 (13)	0.0168 (11)	0.0084 (10)	−0.0057 (11)
C1	0.0620 (17)	0.0566 (15)	0.0439 (15)	−0.0070 (13)	0.0109 (12)	0.0003 (12)
C2	0.0670 (19)	0.0707 (18)	0.0617 (17)	−0.0002 (14)	0.0157 (14)	−0.0015 (14)
C3	0.081 (2)	0.085 (2)	0.080 (2)	0.0071 (17)	0.0337 (18)	−0.0012 (18)
C4	0.119 (3)	0.073 (2)	0.078 (2)	−0.003 (2)	0.056 (2)	−0.0027 (18)
C5	0.119 (3)	0.113 (3)	0.0486 (19)	−0.020 (2)	0.0181 (18)	−0.0134 (17)
C6	0.0767 (19)	0.098 (2)	0.0513 (17)	−0.0100 (16)	0.0084 (14)	−0.0062 (16)
C7	0.0529 (16)	0.0653 (17)	0.0448 (16)	−0.0014 (13)	0.0080 (13)	0.0025 (12)
C8	0.0524 (15)	0.0516 (14)	0.0456 (15)	−0.0068 (12)	0.0121 (12)	−0.0030 (12)
C9	0.0502 (15)	0.0550 (16)	0.0516 (16)	−0.0112 (12)	0.0041 (12)	0.0032 (12)
C10	0.0705 (19)	0.0703 (17)	0.0454 (16)	−0.0090 (15)	0.0058 (13)	0.0030 (13)
C11	0.0694 (18)	0.0566 (16)	0.0486 (16)	−0.0128 (14)	0.0196 (13)	−0.0031 (12)
C12	0.0561 (17)	0.0836 (18)	0.0700 (18)	0.0001 (14)	0.0004 (13)	0.0025 (15)
C13	0.100 (2)	0.0828 (19)	0.0562 (17)	−0.0108 (17)	0.0289 (15)	−0.0094 (15)

### Geometric parameters (Å, °)

**Table:**

O1—C7	1.292 (3)	C4—C5	1.365 (4)
O1—H1	0.820	C4—H4	0.930
O2—C7	1.218 (3)	C5—C6	1.399 (4)
N1—C9	1.336 (3)	C5—H5	0.930
N1—C8	1.350 (3)	C6—H6	0.930
N2—C11	1.326 (3)	C9—C10	1.385 (3)
N2—C8	1.364 (3)	C9—C12	1.494 (3)
N3—C8	1.322 (3)	C10—C11	1.376 (3)
N3—H3A	0.860	C10—H10	0.930
N3—H3B	0.860	C11—C13	1.507 (3)
C1—C6	1.372 (3)	C12—H12A	0.960
C1—C2	1.382 (3)	C12—H12B	0.960
C1—C7	1.489 (3)	C12—H12C	0.960
C2—C3	1.379 (4)	C13—H13A	0.960
C2—H2	0.930	C13—H13B	0.960
C3—C4	1.361 (4)	C13—H13C	0.960
C3—H3	0.930		
			
C7—O1—H1	109.5	O1—C7—C1	114.2 (2)
C9—N1—C8	118.13 (19)	N3—C8—N1	118.5 (2)
C11—N2—C8	116.7 (2)	N3—C8—N2	117.4 (2)
C8—N3—H3A	120.0	N1—C8—N2	124.1 (2)
C8—N3—H3B	120.0	N1—C9—C10	120.4 (2)
H3A—N3—H3B	120.0	N1—C9—C12	116.9 (2)
C6—C1—C2	119.6 (2)	C10—C9—C12	122.7 (2)
C6—C1—C7	119.7 (2)	C11—C10—C9	118.4 (2)
C2—C1—C7	120.7 (2)	C11—C10—H10	120.8
C3—C2—C1	120.3 (3)	C9—C10—H10	120.8
C3—C2—H2	119.9	N2—C11—C10	122.3 (2)
C1—C2—H2	119.9	N2—C11—C13	116.1 (3)
C4—C3—C2	120.1 (3)	C10—C11—C13	121.6 (3)
C4—C3—H3	119.9	C9—C12—H12A	109.5
C2—C3—H3	119.9	C9—C12—H12B	109.5
C3—C4—C5	120.3 (3)	H12A—C12—H12B	109.5
C3—C4—H4	119.8	C9—C12—H12C	109.5
C5—C4—H4	119.8	H12A—C12—H12C	109.5
C4—C5—C6	120.2 (3)	H12B—C12—H12C	109.5
C4—C5—H5	119.9	C11—C13—H13A	109.5
C6—C5—H5	119.9	C11—C13—H13B	109.5
C1—C6—C5	119.4 (3)	H13A—C13—H13B	109.5
C1—C6—H6	120.3	C11—C13—H13C	109.5
C5—C6—H6	120.3	H13A—C13—H13C	109.5
O2—C7—O1	123.4 (2)	H13B—C13—H13C	109.5
O2—C7—C1	122.4 (2)		
			
C6—C1—C2—C3	−2.1 (4)	C9—N1—C8—N3	−178.84 (19)
C7—C1—C2—C3	177.7 (2)	C9—N1—C8—N2	1.2 (3)
C1—C2—C3—C4	−0.3 (4)	C11—N2—C8—N3	178.20 (19)
C2—C3—C4—C5	1.8 (5)	C11—N2—C8—N1	−1.8 (3)
C3—C4—C5—C6	−0.9 (5)	C8—N1—C9—C10	−0.1 (3)
C2—C1—C6—C5	3.0 (4)	C8—N1—C9—C12	179.75 (19)
C7—C1—C6—C5	−176.8 (2)	N1—C9—C10—C11	−0.4 (3)
C4—C5—C6—C1	−1.5 (4)	C12—C9—C10—C11	179.9 (2)
C6—C1—C7—O2	−5.3 (4)	C8—N2—C11—C10	1.4 (3)
C2—C1—C7—O2	175.0 (2)	C8—N2—C11—C13	−178.11 (19)
C6—C1—C7—O1	174.4 (2)	C9—C10—C11—N2	−0.3 (4)
C2—C1—C7—O1	−5.3 (3)	C9—C10—C11—C13	179.1 (2)

### Hydrogen-bond geometry (Å, °)

**Table:**

*D*—H···*A*	*D*—H	H···*A*	*D*···*A*	*D*—H···*A*
O1—H1···N1	0.82	1.82	2.606 (2)	160
N3—H3A···O2	0.86	2.16	3.003 (3)	168
N3—H3B···N2^i^	0.86	2.25	3.098 (3)	169

Symmetry codes: (i) −*x*, −*y*+2, −*z*+1.
